# DR1440 is a potential iron efflux protein involved in maintenance of iron homeostasis and resistance of *Deinococcus radiodurans* to oxidative stress

**DOI:** 10.1371/journal.pone.0202287

**Published:** 2018-08-14

**Authors:** Shang Dai, Ye Jin, Tao Li, Yulan Weng, Xiaolin Xu, Genlin Zhang, Jiulong Li, Renjiang Pang, Bing Tian, Yuejin Hua

**Affiliations:** 1 Key Laboratory for Nuclear-Agricultural Sciences of Chinese Ministry of Agriculture and Zhejiang Province, Institute of Nuclear-Agricultural Sciences, Zhejiang University, Hangzhou, China; 2 Key Laboratory for Green Processing of Chemical Engineering of Xinjiang Bingtuan, School of Chemistry and Chemical Engineering, Shihezi University, Shihezi, Xinjiang, China; East Carolina University Brody School of Medicine, UNITED STATES

## Abstract

Iron acquisition by bacteria is well studied, but iron export from bacteria is less understood. Herein, we identified *dr1440* with a P-type ATPase motif as a potential exporter of iron from *Deinococcus radiodurans*, a bacterium known for its extreme resistance to radiation and oxidants. The DR1440 was located in cell membrane as demonstrated by fluorescence labelling analysis. Mutation of *dr1440* resulted in cellular accumulation of iron ions, and expression level of *dr1440* was up-regulated significantly under iron ion or hydrogen peroxide stress in the wild-type strain, implicating DR1440 as a potential iron efflux protein. The *dr1440* mutant displayed higher sensitivity to iron ions and oxidative stresses including hydrogen peroxide, hypochlorous acid, and gamma-ray irradiation compared with the wild-type strain. The high amount of iron in the mutant strain resulted in severe protein carbonylation, suggesting that DR1440 might contribute to intracellular protein protection against reactive oxygen species (ROS) generated from ferrous ion-mediated Fenton-reaction. Mutations of S297A and C299A led to intracellular accumulation of iron, indicating that S297 and C299 might be important functional residues of DR1440. Thus, DR1440 is a potential iron efflux protein involved in iron homeostasis and oxidative stress-resistance of *D*. *radiodurans*.

## Introduction

Iron (Fe) ions are essential nutrients required for microorganisms and act as important enzyme cofactors for a number of cellular processes such as DNA synthesis and repair, antioxidant systems and electron transport [1]. However, these ions are harmful to cells when in excess due to Fenton-reaction which generates harmful hydroxyl radicals (HO·) that target DNA, RNA, proteins and lipids [2]. Iron acquisition and efflux systems play important roles in iron homeostasis and intracellular redox-cycling processes in bacteria. Several bacterial iron efflux transporters have been documented including P1B-type ATPases such as *Bacillus subtilis* PfeT, *Listeria monocytogenes* FrvA, group A *Streptococcus* PmtA and *Sinorhizobium meliloti* Nia, major facilitator superfamily (MFS) proteins such as *Salmonella typhimurium* IceT, and membrane bound ferritin-like proteins such as *Agrobacterium tumefaciens* MbfA [3]. *Escherichia coli* YiiP (FieF) was supposed to be an iron exporter belonging to cation diffusion facilitator (CDF) family [3]; however, the role of YiiP and its eukaryotic homologs were challenged and suggested to be a Zn^2+^ exporter [4, 5]. Iron exporters and iron homeostasis in prokaryotes are far from being understood, especially in extremophiles.

*Deinococcus radiodurans* is an extremophilic bacterium known for its resistance to stresses including ionizing radiation (IR), ultraviolet (UV) radiation, desiccation and oxidative stress [6–8]. *D*. *radiodurans* demonstrates remarkable resistance to oxidative stress incurred from reactive oxygen species (ROS), which are generated upon exposure to radiation and oxidants [9, 10]. Cellular defence against protein damage by ROS is proposed to be crucial in the stress-resistance of *D*. *radiodurans* [11], the genome of which codes numerous Fe^2+^-acquisition genes including an ABC-type hemin transporter (*drb0016*), an ABC-type Fe(III)-siderophore transporter (*drb0017*), and two Fe(II) transporters (*dr1219* and *dr1220*) [2]. Nevertheless, iron exporters in *D*. *radiodurans* remain unclear.

DR1440 is a putative P1B-ATPase subfamily member in *D*. *radiodurans*. P-type ATPases constitute a large protein family that pump ions or lipids across cellular membranes [12]. In general, P-type ATPases contain five functional and structurally distinct domains (three cytoplasmic domains and two membrane-embedded domains). P1B-ATPases are commonly responsible for transporting heavy metals such as Cu, Zn and Fe [3, 13].

In the present study, we identified DR1440 as a potential P-type iron efflux protein in *D*. *radiodurans*, and demonstrated that it might play an important role in maintaining intracellular Fe homeostasis. The mutation of DR1440 led to the high cellular sensitivity to iron ions and oxidative stress, and the increased intracellular protein carbonylation. Our findings revealed that amino acids Ser297 and Cys299 may be functionally important to DR1440.

## Materials and methods

### Bacterial strains and growth media

All strains and plasmids used in this study are listed in Table A in S1 File. *Escherichia coli* strains were grown in Luria-Bertani (LB) broth medium (1% tryptone, 0.5% yeast extract, and 1% sodium chloride) with aeration or on LB agar plates (1.2% Bacto-agar) at 37°C supplemented with appropriate antibiotics. All *D*. *radiodurans* strains were grown at 30°C in TGY medium (0.5% tryptone, 0.1% glucose, and 0.3% yeast extract) with aeration or on TGY plates supplemented with 1.5% Bacto-agar.

### Sequence alignment and homology modeling

Protein sequences of previously characterized P-type cation-transporting ATPases from *Shigella sonnei*, *Bacillus subtilis*, *Legionella pneumophila*, *Escherichia coli* and *Thermus thermophilus* were obtained from the NCBI database (https://www.ncbi.nlm.nih.gov/). Sequence alignment of these P-type ATPases with DR1440 from *D*. *radiodurans* was performed with the CLUSTALW software (http://www.genome.jp/tools-bin/clustalw) (Fig A in S1 File). The protein structure of DR1440 was predicted by homology modelling using Phyre2 (http://www.sbg.bio.ic.ac.uk/phyre2) with copper efflux ATPase (PDB:3RFU) as a starting model. Structural representations were generated using PyMOL (http://www.pymol.org/).

### Construction of mutant strains

Tripartite ligation and double-crossover recombination methods were used for gene mutation as described previously [14] with some modifications. Briefly, upstream and downstream fragments of the target gene were amplified using primers with *Bam*HI and *Hind*III restriction enzyme sites, respectively. After digestion, fragments were ligated to a streptomycin resistance cassette from pMD18-T and the amplified ligation product was transformed into *D*. *radiodurans* (Fig B in S1 File). The mutant strain in which the target gene was replaced with the streptomycin resistance fragment was screened using streptomycin. Primers used in this study are listed in Table B in S1 File. Primers P1 and P2 were used to amplify a 488 bp DNA fragment upstream of the targeted gene with a *Bam*HI restriction site, and primers P3 and P4 were used to obtain a 460 bp fragment downstream of the targeted genes with a *Hind*III restriction site. These two fragments were digested with *Bam*HI and *Hind*III, respectively, and ligated to the streptomycin-resistant DNA fragment pretreated with the same enzymes. The ligation product was amplified by PCR using primers P1 and P4. The PCR product was then purified using a Wizard SV Gel and PCR Clean-up System kit (Promega Co., USA) and transformed into *D*. *radiodurans* R1 strain treated with CaCl_2_. Mutant colonies were selected on TGY plates containing 10 μg/mL streptomycin. Null mutants were confirmed by PCR product size and DNA sequencing. Primers P5 and P6 (Table B and Fig B in S1 File) were used for detection of the interior fragment (NC_001263.1: c1444754-1445364) of the *dr1440*. The resulting mutant was designated as Mt-1440.

### Complementation of the gene mutant and site-directed mutagenesis of *dr1440*

For complementation of the mutant, genomic DNA was isolated from the wild-type R1 strain. A 1650-bp region containing the *dr1440* gene was amplified with primers *dr1440*_c_ forward and *dr1440*_c_ reverse (Table B in S1 File) and ligated to the pRADK vector to obtain pRAD-*dr1440*, which was subsequently transformed into the *dr1440* null mutant to yield gene complementation strain Mt-1440C.

Site-directed mutagenesis of *dr1440* was performed using the Quick Change Site-directed Mutagenesis Kit (Stratagene Co., USA) following the manufacturer's protocol. Briefly, the fragment containing the mutated sequence was cloned into the shuttle vector and transformed into *D*. *radiodurans* as described previously [15]. The *dr1440* sequence amplified using the corresponding site-directed mutagenesis primers and pRAD-*dr1440* as a template (Tables A and B in S1 File) were treated with *Dpn*I to digest the methylated vector template. Following digestion, DNA fragments were cloned into the pRADK shuttle vector. S297A, P298A, and C299A site mutations in the plasmids pRAD-S297A, pRAD-P298A, pRAD-C299A were confirmed by DNA sequencing, and these constructs were transformed into the *dr1440* null mutant, respectively. Gene complementation and site-directed mutagenesis strains were confirmed by PCR and DNA sequence analysis.

### Real-time quantitative PCR

Real-time quantitative PCR (qRT-PCR) was used to measure *dr1440* gene expression under different stress conditions. First, cells were grown to OD_600_ = 0.6 and treated with 30–60 mM H_2_O_2_, 1 mM MnCl_2_ or FeCl_2_ for 1h, respectively. Cells were then harvested by centrifugation at 5000 *g* at 4°C. Total RNA was extracted from 5 mL of cell cultures using TRIZOL reagent (Invitrogen Corp., Carlsbad, CA, USA). cDNA synthesis was carried out in 20 μL reaction mixtures containing 1 μg of each DNase I-treated and purified total RNA sample, and 3 μg of random hexamers. The qRT-PCR experiments were performed using SYBR Premix Ex Taq (TaKaRa Biotechnology Ltd, Japan). Primers used for qRT-PCR are listed in the Table B in S1 File. Differences in relative transcript abundance level were calculated and indicated by 2^-ΔΔCt^ [15]. Gene *dr1343* encoding glyceraldehyde 3-phosphate dehydrogenase (GADPH) was used as an internal control. All assays were performed using the STRAGENE Mx3005P Real-time detection system.

### Western blot analysis

Protein expression levels were confirmed using western blotting as described previously [15]. Briefly, a 6 × His tag was fused to the C-terminal of DR1440 using the tripartite ligation and double-crossover recombination method as shown in Fig B in S1 File. Monoclonal anti-6 × His mouse antibody (Proteintech, USA) was used to detect DR1440-6 × His. Horseradish peroxidase-conjugated goat anti-mouse and anti-rabbit IgG were added as secondary antibodies. The expression level of GroEL detected by a rabbit anti-GroEL polyclonal antibody served as an internal control (Sigma, USA). The commercial anti-GroEL can be used to detect GroEL in *D*. *radiodurans* [16].

### Cellular localization of DR1440

To confirm the localization of the DR1440 protein, expression of *dr1440* fused to the enhanced green fluorescence protein (eGFP) gene was analyzed by fluorescence microscopy as described previously [17]. Plasmid pRADG-*dr1440* was constructed by cloning the target gene into the corresponding sites of pRADG containing the eGFP gene (BD Clontech, USA) under the control of the *groEL* promoter (PgroEL). The pRADG-*dr1440* construct was transformed into *D*. *radiodurans* wild type R1 strain using the CaCl_2_ technique. Plasmid pRADG without the target gene was transformed into the wild type as a negative control. The transformant was obtained using chloramphenicol resistance selection, and verified by DNA sequencing. The transformant was grown to exponential phase (OD_600_ ~1.0), spread on a glass slide and examined by using a Zeiss LSM510 laser confocal microscope. To differentiate cell membrane and nucleoid, nucleoid in the transformant was stained using DAPI and blue fluorescence was analysed.

### Intracellular Fe, Mn, Zn, and Cu ions assays

Intracellular concentrations of metal ions were determined following the methods reported previously [18]. Bacterial cells were cultured in 500 mL TGY broth pretreated with Chelex to remove any cations. Cultures were then supplemented with metal ion mixture containing 50 μM each of manganese chloride, zinc chloride, copper chloride, and ferrous chloride. The cells grown to OD_600_ = 1.0, harvested by centrifugation at 10,000 *g*, 4°C for 10 min, and pellets were washed three times with 1 × phosphate-buffered saline (PBS, pH 7.5) containing 1 mM EDTA, rinsed three times with 1 × PBS containing no EDTA, and cells were freeze-dried for 24 h. For metal ion analysis, 5 mL Ultrex II nitric acid (Fluka AG., Buchs, Switzerland) and 1 mL H_2_O_2_ were added to the dried cells and incubated at 100°C for 2 h. The metal ion concentration in samples was measured using inductive coupled plasma mass spectrometry (ICP-MS: ELAN DRC-e, PerkinElmer, USA). A control was prepared in the same manner but without metal ion treatment. All data are represented as means and standard deviation of three independent experiments (mean ± SD).

### Metal cation sensitivity assays

Metal cation sensitivity assays were carried out as described previously [18]. Separate 1 M solutions of manganese chloride, cobalt chloride, nickel chloride, zinc chloride, copper chloride and ferrous chloride (Sigma) were prepared in Milli-Q water and filter-sterilized by passing through 0.22-μm filter. Cells of the wild-type R1, *dr1440* mutant (Mt-1440), and its gene complementation (Mt-1440C) strains were grown to OD_600_ ~1.0 and plated on TGY plates with 5 mm sterile discs containing 1 M solutions of various cations. Plates were incubated for 3 days and inhibition zones on each disc were measured.

To evaluate the effect of iron ions on the growth of Mt-1440 and the wild-type R1 cells, FeCl_2_ solutions with increasing concentrations were added to the cell cultures, and 200 μL of diluted cultures were plated on TGY plates and incubated at 30°C for 3 days. Colonies were counted. All data are represented as means and standard deviation of at least three independent experiments (mean ± SD).

### Survival assays

*D*. *radiodurans* wild-type R1 and mutant strains were cultured in TGY broth to OD_600_ = 0.8, centrifuged, and resuspended in PBS buffer. A 100 μL sample of cell suspension was diluted with PBS to 10^7^ colony-forming units (CFU) mL^-1^. Survival assays under H_2_O_2_, HClO and irradiation treatments were performed as described previously [15]. For H_2_O_2_ and HClO treatments, the cell suspensions were treated with different concentrations of H_2_O_2_ or HClO for 30 min, and cells were then plated and cultured on TGY plates for 3 days before colonies were counted. For the irradiation treatment, the cell suspension was irradiated with different doses of ^60^Co γ-ray for 1 h on ice. Different doses were achieved by adjusting the sample distance from a γ-ray source. After irradiation treatment, cells were plated and incubated on TGY plates at 30°C for 3 days, and colonies were counted. All data are represented as mean ± SD from at least three independent experiments.

### Intracellular ROS accumulation assays

ROS accumulation assays were performed using 2', 7'-dichlorofluorescein diacetate (DCFH-DA) as a molecular probe [19]. A 2 mL sample of cell cultures (OD_600_ = 0.8) were washed three times with PBS, and pellets were resuspended in DCFH-DA and incubated at 37°C for 30 min. After incubation, cells were washed three times with PBS and resuspended in 2 mL PBS. A 1 mL sample was then treated with or without 50 mM H_2_O_2_ for 30 min. DCFH-DA is hydrolyzed into DCFH by esterase then oxidized by intracellular ROS into DCF, which produces fluorescence that can be measured using a fluorescence spectrophotometer (SpectraMax M5) at an excitation wavelength of 485 nm and an emission wavelength of 525 nm [19].

### Protein carbonylation assays

Protein carbonylation assays were carried out as described previously [20, 21]. A 1 mL sample of cell cultures grown to OD_600_ = 1.0 was treated with 50 mM H_2_O_2_ for 30 min, harvested, and resuspended in PBS containing 1% (by volume) β-mercaptoethanol and 1 mM phenylmethanesulfonyl fluoride. A 1mL sample of cells not treated with H_2_O_2_ was used as a negative control. Cells were disrupted by sonication and protein concentration in the cell-free extract was determined by the Bradford method. Protein carbonylation, an indicator of protein oxidation, was measured using western blot assays and the 2,4-dinitrophenyl hydrazine (DNPH) spectrophotometric method, respectively. For western blot assays, protein carbonylation in the extract (4 mg total protein/mL) was identified using an OxyBlot Protein Oxidation Detection Kit (S7150) (Merck Co., USA) as described previously [21]. A 5 μL sample of protein lysate was mixed with 5 μL of 12% sodium dodecyl sulphate (SDS) and 10 μL DNPH and incubated at room temperature for 15 min. After incubation, 7.5 μL of neutralization reagent was added. The sample was loaded onto 12% Bis-Tris gels for separation. The gel was then transferred to a PVDF membrane for 25min at 10V. The membrane was then incubated with primary antibody specific to the DNPH moiety attached to the proteins. Then the membrane was incubated with a horseradish peroxidase peroxidase-conjugated antibody directed against the primary antibody. After treated with chemiluminescent substrate, the membrane was imaged by exposure to light-sensitive films.

For DNPH spectrophotometric assays [20], the cell-free extracts were incubated with 400 μL of 10 mM DNPH in 2 M HCl for 2 h in the dark. After precipitation using 10% trichloroacetic acid at 4°C, the precipitated proteins were washed three times with 50% ethyl acetate in ethanol. After evaporation, decolorized protein precipitates were dissolved in 1 mL of 6 M guanidine hydrochloride, the solution was centrifuged, and the absorbance of the supernatant was determined at 370 nm against a protein control that had been processed in parallel but with 2 M HCl instead of DNPH. The protein carbonyl content was defined as mM/μg protein.

### Statistical analysis

Student’s *t*-tests were used to assess the significance between results, and *p* < 0.05 was considered significant.

## Results

### Identification of a potential iron efflux protein located in the cell membrane of *D*. *radiodurans*

**Fig 1:**
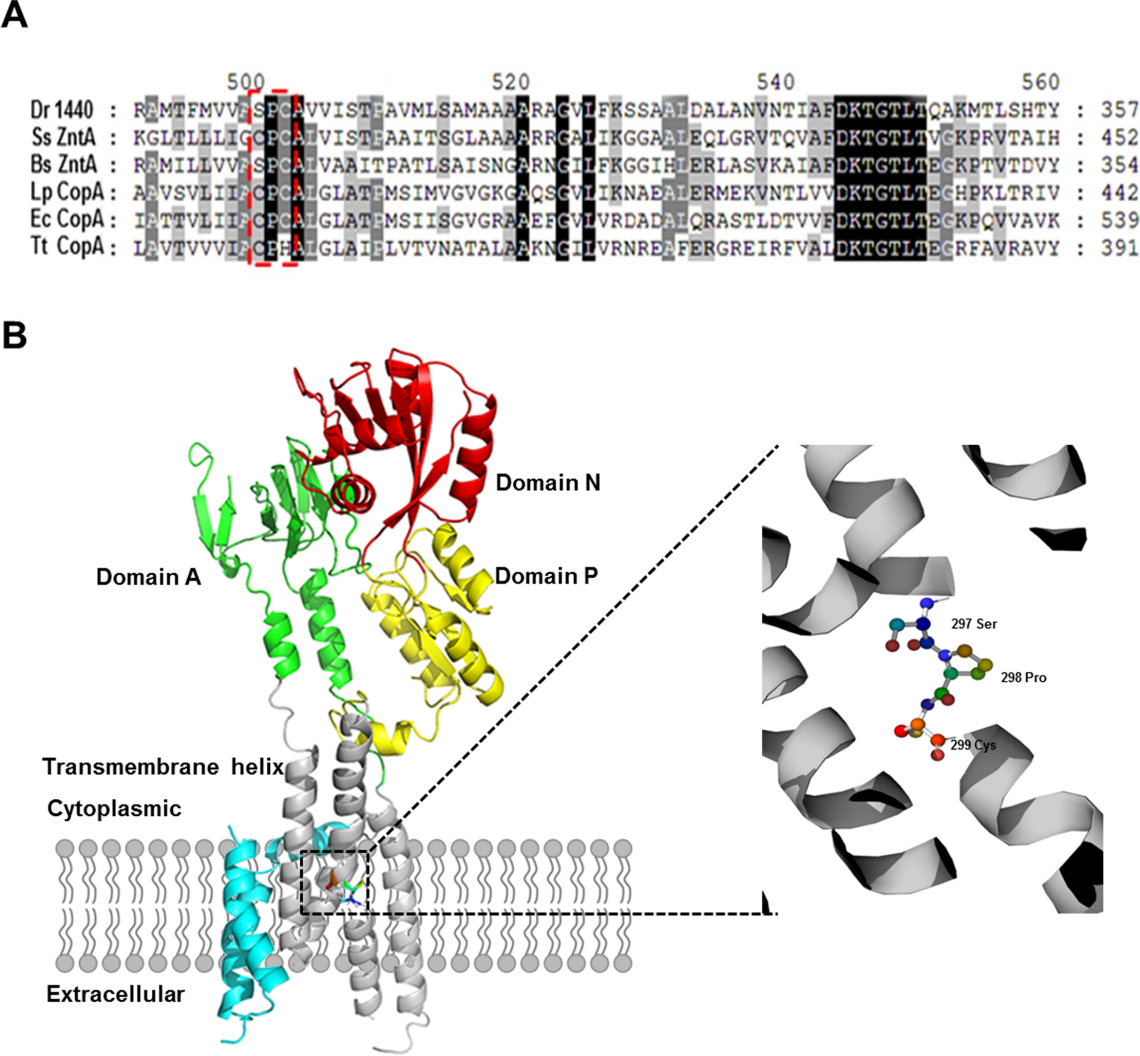
Comparison of DR1440 and P-type ATPase homologs. (A) Multiple sequence alignments of DR1440 from *D*. *radiodurans* with P-type ATPase homologs. Sequences are from following bacteria: DR1440 from *D*. *radiodurans*, SsZntA from *Shigella sonnei*, BsZntA from *Bacillus subtilis*, LpCopA from *Legionella pneumophila*, EcCopA from *Escherichia coli* and TtCopA from *Thermus thermophilus*. The conserved metal binding sequence SPC is indicated by a dashed line box. Identical residues are shown as white letters with black background, and similar residues are shown as white letters with gray background. (B) The predicted structure of DR1440 obtained from homology modelling. The left panel shows the predicted structure of DR1440. Domain A is a Actuator domain, which contains a signature motif Thr-Gly-Glu (TGE) of P-type ATPases. Domain P contains conserved DKTG sequence related to the hydrolysis of ATP. Domain N performs ATP binding and phosphorylates the P-domain. The transmembrane (TM) domain consists of six membrane-spanning segments and harbours the metal ion binding sites. The right panel shows a close-up view of the TM helix containing the SPC metal binding site. CopA from *L*. *pneumophila* was used as a starting model.

The hypothetical gene *dr1440* in *D*. *radiodurans* was predicted to encode a metal translocating P-type ATPase by the BlastP program (http://www.ncbi.nih.nlm.gov). The gene shares 30% sequence identity with a P-type ATPase from *Legionella pneumophila*. DR1440 contains the Thr-Gly-Glu (TGE) signature motif of P-type ATPases (Fig A in S1 File) and a putative metal binding sequence SPC (Fig 1A), which conserved in heavy-metal pumps (PIB-type ATPases) [22]. PIB-type ATPases exist in all life forms and are the most common P-type ATPases in bacteria and archaea. They transport heavy metals across biomembranes, and play a key role in homeostasis and tolerance of heavy metals [13]. The protein structure of DR1440 was predicted using the crystal structure of CopA from *Legionella pneumophila* as a model, suggesting that the SPC site was located in the transmembrane helix region (Fig 1B).

**Fig 2:**
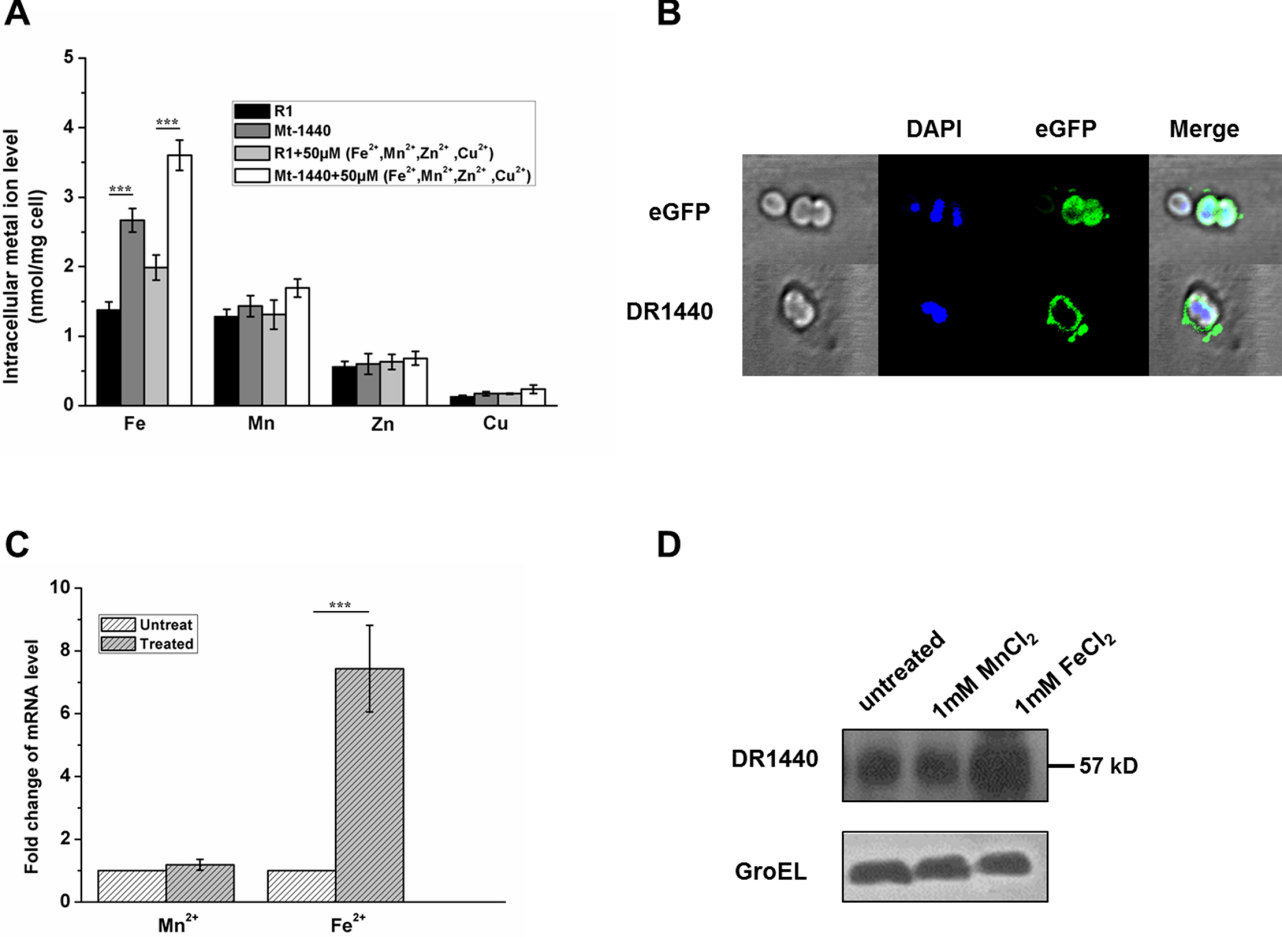
DR1440 is a potential iron efflux protein located in the cell membrane. (A) Intracellular concentration of metal ions in wild-type R1 and mutant Mt-1440 strains cultured in TGY medium supplemented with or without metal ion mixture containing 50 μM each of Fe^2+^, Mn^2+^, Zn^2+^ and Cu^2+^. R1, the wild type strain; Mt-1440, *dr1440* mutant. ***, *p*<0.001. (B) Localization of DR1440 using fluorescence labelling analysis. The *dr1440* was fused to the eGFP gene and ligated into the pRADG plasmid, and the resulting construct was transformed into wild-type R1 cells. Plasmid pRADG without target gene was transformed into the wild-type strain as a negative control. The eGFP expressed in *D*. *radiodurans* R1 was localized in cytoplasm. All transformants were spread on glass slides and examined with a laser confocal microscope. Nucleoids were stained with DAPI (blue fluorescence). Merged images indicated the co-localization of the eGFP-labelled protein in the cell membrane. (C) mRNA level of *dr1440* with 1 mM Fe^2+^ or Mn^2+^ treatment, compared with untreated controls. (D) Protein expression level of DR1440 with 1 mM Fe^2+^ or Mn^2+^ treatment, compared with untreated controls. A monoclonal anti-6 × His mouse antibody (Proteintech, USA) was used to detect the 6 × His tag fused to the C-terminal of DR1440. GroEL was used as a control, and detected using anti-GroEL antibody under the same treatment conditions used for DR1440.

A gene knockout mutant of *dr1440*, designated as Mt-1440, was constructed using a streptomycin-resistance gene replacement strategy (Fig B in S1 File). The metal ion content in mutant cells grown in media supplemented with metal ion mixture containing 50 μM each of FeCl_2_, MnCl_2_, ZnCl_2_ and CuCl_2_ was measured by ICP-MS and compared with that of the wild type strain. The iron ion level in the mutant was approximately 1.5-fold higher than in the wild-type R1 strain in the absence or presence of supplemented metal ions (Fig 2A), indicating that the *dr1440* mutation resulted in intracellular accumulation of iron. However, the intracellular level of other metals including Mn, Zn or Cu in the mutant was similar to that in the wild type strain (Fig 2A), suggesting that DR1440 might be a potential iron efflux protein in *D*. *radiodurans*.

To determine the cellular localization of DR1440, the *dr1440* fused to the eGFP gene was expressed in *D*. *radiodurans* and analyzed using a laser confocal fluorescence microscope. The eGFP control was localized in the cytoplasm, whereas the transformant expressing *dr1440-*eGFP exhibited green fluorescence only in the cell membrane (Fig 2B), indicating that DR1440 was localized in the cell membrane.

Expression levels of *dr1440* in the wild-type R1 strain were analyzed in the presence of supplemented iron ions using qRT-PCR and western blot assays. The mRNA level of *dr1440* was significantly up-regulated under 1 mM FeCl_2_, while no obvious change was detected under 1 mM MnCl_2_ (Fig 2C). Western blot assays showed that the expression level of DR1440 was upregulated under 1 mM FeCl_2_ compared with that in untreated cells or cells treated with 1mM MnCl_2_ (Fig 2D), suggesting that DR1440 was regulated in response to iron stress.

### Mutation of *dr1440* increases iron ion sensitivity

**Fig 3:**
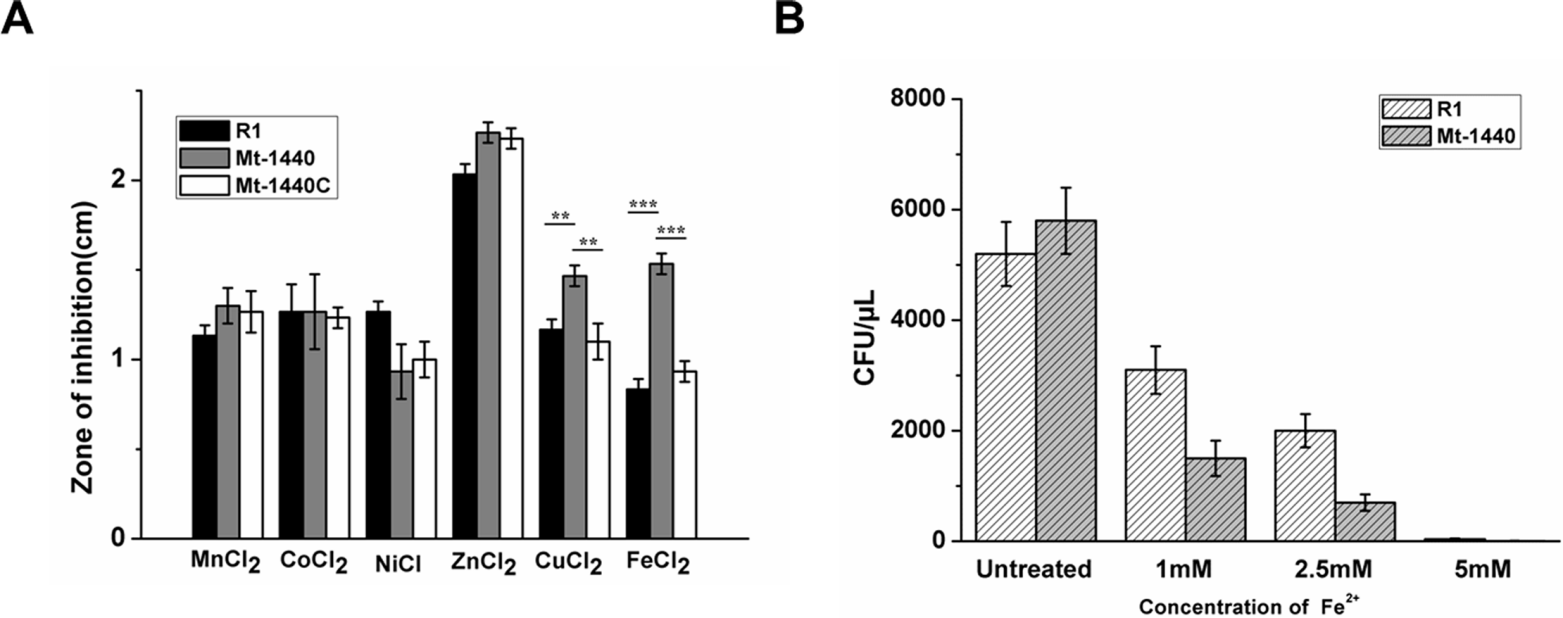
Iron ion sensitivity of wild-type, mutant and gene complementation strains. (A) Zone of inhibition of wild-type (R1), mutant Mt-1440 (Mt-1440) and gene complementation strain (Mt-1440C) under various metal cation stresses. Cells were cultured on TGY plates overlaid with filter discs saturated with 1 M solutions of various cations. **, *p* < 0.01;***, *p* < 0.001. (B) Cell growth of wild-type and Mt-1440 strains following treatment with Fe^2+^ at different concentrations. Cell growth is presented as colony forming units (CFU) per μL of cell cultures.

Supplementation with metal ions can inhibit the growth of an exporter mutant; hence, metal ion sensitivity assays were performed to verify certain metal ion exporter [20]. The metal ion sensitivity of the wild type, Mt-1440, and gene complementated strain (Mt-1440C) was analyzed (Fig 3A). The growth of Mt-1440 was strongly inhibited by iron and copper ions compared with the wild-type and Mt-1440C cells, indicating that mutation of *dr1440* affected intracellular metal homeostasis and led to increased cell sensitivity to external iron ions or copper ions. We could not rule out the possibility that DR1440 might be involved in the export of copper; but mutation of *dr1440* did not result in intracellular accumulation of Cu ions (Fig 2A).

To confirm the iron ion sensitivity of Mt-1440 cells, we measured the effect of various concentrations of iron ions on the growth of Mt-1440 (Fig 3B). Compared with wild-type, Mt-1440 cells exhibited more pronounced defective growth in the presence of increasing concentrations of iron ions. Similarly, a mutant *Streptococcus pneumoniae* strain with a disrupted calcium efflux system exhibited severely decreased cell growth in the presence of high concentrations of calcium ions [23]. Thus, the observed iron ion sensitivity of the DR1440 mutant might be due to cellular deficiency in exporting excess intracellular iron ions, resulting in accumulation of toxic ROS generated via iron ion-mediated Fenton-reaction. These results further supported the role of DR1440 as a potential iron efflux protein involved in the maintenance of iron homeostasis.

### DR1440 is involved in resistance to oxidative stresses

**Fig 4:**
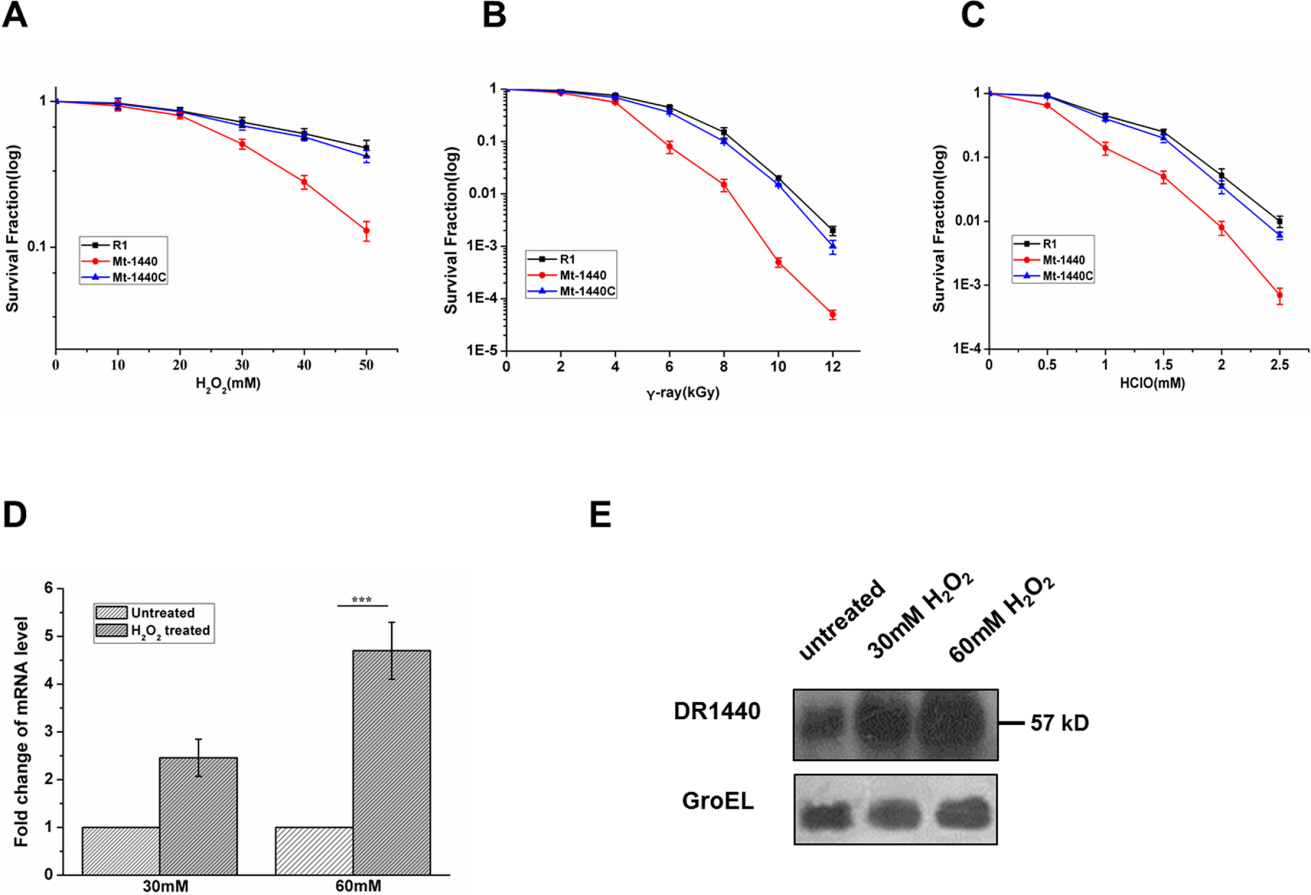
DR1440 is involved in resistance to oxidative stress in *D*. *radiodurans*. (A–C) Survival curves of *D*. *radiodurans* strains following exposure to H_2_O_2_, γ-ray radiation, and HClO. Cell cultures at OD_600_ = 0.8 were diluted with phosphate-buffered saline (PBS) to 10^7^ colony-forming units (CFU) ml^-1^, and treated with H_2_O_2_ or HClO for 30 min, or γ-rays for 1 h. Cells were then plated and cultured on TGY plates for 3 days before colonies were counted. R1, wild-type strain; Mt-1440, DR1440 mutant; Mt-1440C, DR1440 gene complementation strain. (D) Relative mRNA level of *dr1440* in the presence or absence of H_2_O_2_ as assayed by qRT-PCR. ***, *p* < 0.001. (E) Protein expression levels of DR1440 in the presence or absence of H_2_O_2_ measured using western blotting. GroEL was used as a control, and detected using anti-GroEL antibody under the same treatment conditions used for DR1440.

In general, excessive Fe^2+^ *in vivo* will cause serious ROS generation via Fenton-reaction especially when cells suffer from oxidative stress [1]. Cell survival experiments under stress induced by H_2_O_2_, HClO, or γ-ray radiation were performed to evaluate whether DR1440 could contribute to cellular stress-resistance. Mt-1440 cells displayed increased sensitivity to H_2_O_2_, HClO, or γ-ray radiation, whereas the gene complemented Mt-1440C strain exhibited a survival phenotype comparable with wild-type R1 cells (Fig 4A–4C), suggesting that the potential iron exporter may be involved in resistance to oxidative stress in *D*. *radiodurans*.

Expression of DR1440 in wild-type cells under H_2_O_2_ stress was analyzed using qRT-PCR and western blot assays. The mRNA level of DR1440 gene was significantly up-regulated following treatment with 30 mM and 60 mM H_2_O_2_ (Fig 4D), consistent with previous results using RNA-sequence analysis showing that *dr1440* was up-regulated under treatment with 100 mM H_2_O_2_ [15]. Western blot assay verified the expression profiles of DR1440 under hydrogen peroxide stress (Fig 4E), confirming that DR1440 was induced by hydrogen peroxide. These results indicated that DR1440 might play an important role in resistance to peroxide stress in *D*. *radiodurans*.

### ROS generation in Mt-1440 leads to protein carbonylation

**Fig 5:**
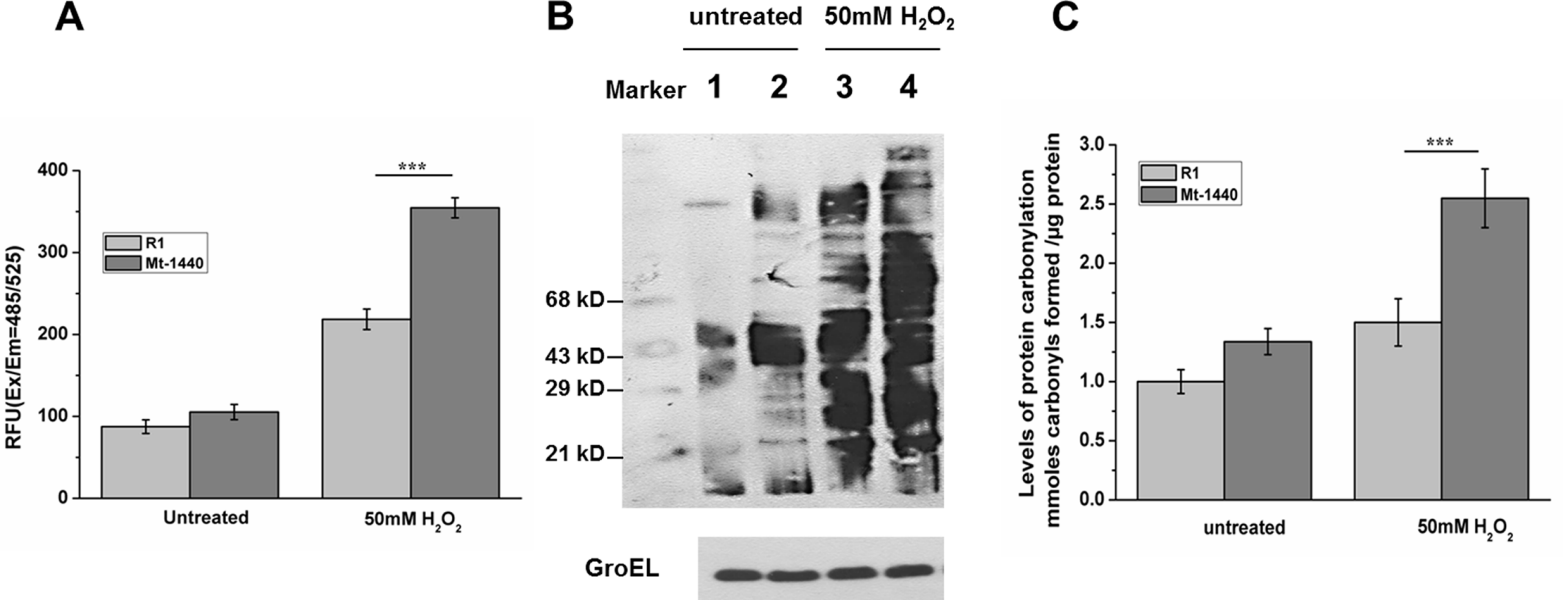
ROS accumulation in Mt-1440 mutant cells leads to protein carbonylation. (A) ROS accumulation in wild-type R1 and Mt-1440 mutant cells in the presence or absence of 50 mM H_2_O_2_ treatment. ***, *p* < 0.001. (B) Protein carbonylation in wild-type R1 and Mt-1440 cells detected using western blotting. Cells (OD_600_ = 1.0) were harvested and treated with or without 50 mM H_2_O_2_ for 30 min. Lane 1, untreated wild-type R1 cells; Lane 2, untreated Mt-1440 cells; Lane 3, wild-type R1 cells treated with H_2_O_2_; Lane 4, Mt-1440 cells treated with H_2_O_2_. GroEL was used as a protein loading control and detected by an anti-GroEL antibody (C) Protein carbonylation levels in wild-type R1 and Mt-1440 cells measured using DNPH spectrophotometric assays. Cells (OD_600_ = 1.0) were harvested and treated with or without 50 mM H_2_O_2_ for 30 min. ***, *p* < 0.001.

Proteins are the critical targets in cells under oxidative stress and the level of protein carbonylation is an important index of intracellular oxidative damage to proteins [24]. Mt-1440 cells accumulated 1.8-fold more ROS than wild-type R1 cells following H_2_O_2_ treatment (Fig 5A), consistent with the observed increase in intracellular protein carbonylation under H_2_O_2_ stress as detected using western blot assays (Fig 5B). Moreover, DNPH spectrophotometric assays showed that protein carbonylation levels were ~1.7-fold higher in Mt-1440 cell than in wild-type cells under H_2_O_2_ stress (Fig 5C), indicating that mutation of *dr1440* resulted in a higher level of protein oxidation than in wild-type cells. This suggested that the potential iron efflux protein could help to maintain relative low levels of intracellular iron, and thereby enhance cellular resistance by protecting proteins against oxidative damage.

### Effects of site mutations of DR1440 on intracellular accumulation of iron and oxidative stress-resistance of *D*. *radiodurans*

**Fig 6:**
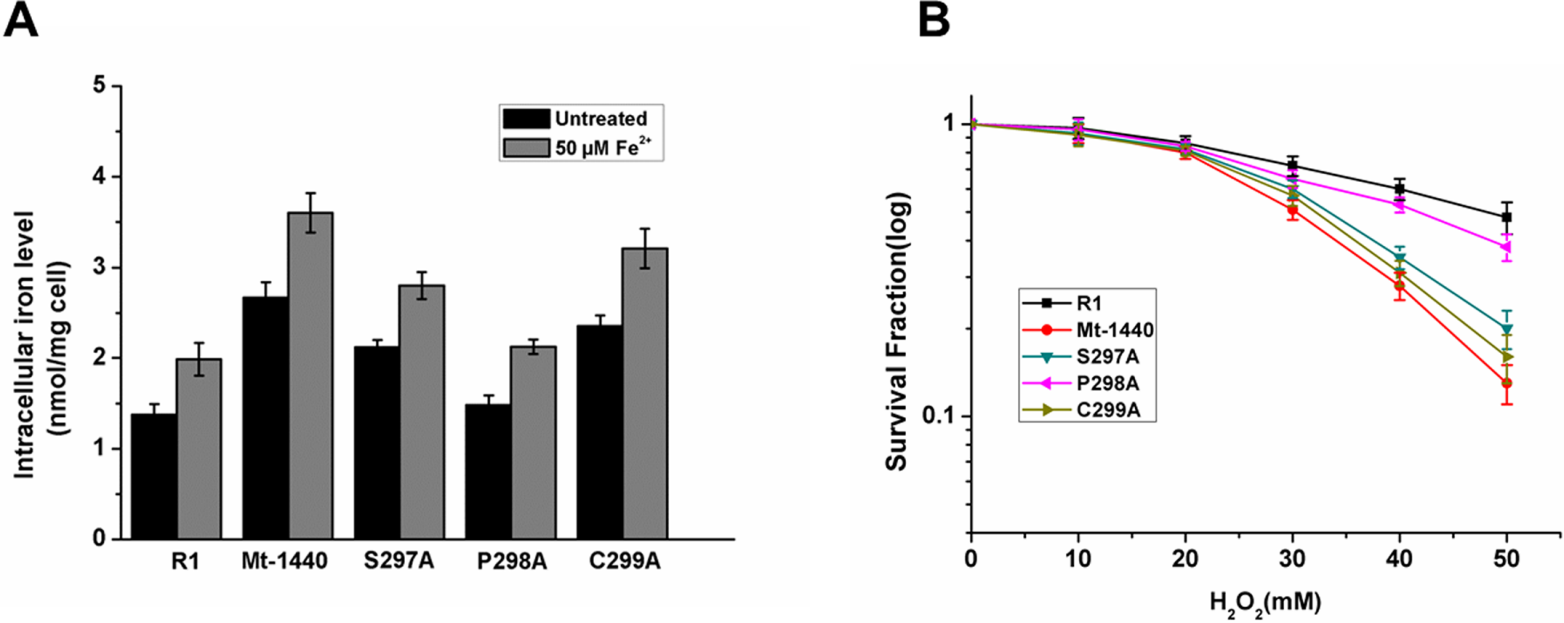
Effects of DR1440 site mutations on intracellular accumulation of iron and oxidative-stress resistance of *D*. *radiodurans*. (A) Intracellular iron ion concentrations in wild-type R1, mutant (Mt-1440), and site mutants of DR1440 (S297A, P298, and C299A) treated with or without 50 μM Fe ions (FeCl_2_). (B) Survival fractions for wild-type R1, *dr1440* mutant (Mt-1440) and site mutants (S297A, P298, and C299A) under H_2_O_2_ stress. Cell cultures at OD_600_ = 0.8 were diluted with PBS to 10^7^ CFU ml^-1^ and treated with H_2_O_2_ for 30 min. Cells were then plated and cultured on TGY plates for 3 days before the colonies were counted.

A putative metal binding motif SPC was identified in DR1440 (Fig 1A). To evaluate the roles of these conserved residues in the function of DR1440, we constructed site mutants (S297A, P298A, and C299A), in which the amino acid residues S297, P298, and C299 were replaced with alanine (A). S297A and C299A mutations resulted in intracellular accumulation of Fe, similar to the null mutant Mt-1440 in the absence or presence of supplemented iron ions, while the P298A mutation did not affect the intracellular concentration of Fe (Fig 6A). This indicated that S297 and C299 might be important residues for metal binding in DR1440. Moreover, S297A or C299A mutations resulted in increased cell sensitivity to H_2_O_2_ similar to Mt-1440 mutant strain, while the P298A mutation had little effect on cell survival under H_2_O_2_ stress (Fig 6B).

## Discussion

DR1440 was identified as a potential iron efflux protein in the extremophilic bacterium *D*. *radiodurans*, known for its resistance to oxidative stress. Iron is an essential metal for many cellular functions including N_2_ fixation, photosynthesis, respiration, trichloroacetic acid (TCA) cycle, oxygen transport, and DNA biosynthesis [25, 26]. However, excessive accumulation of iron ions is toxic to the cells because it leads to the generation of ROS via Fenton-reaction. Recently, an iron efflux system was identified in *Bradyrhizobium japonicum*, of which the N-terminal domain belongs to the ferritin-like AB protein family, and the C-terminal domain is similar to CCC1 in eukaryotes [27]. However, iron exporters in prokaryotes are poorly understood, especially in extremophilic bacteria. In the present study, evidence from several lines indicated that DR1440 functions as a potential iron efflux protein. First, DR1440 contained a P-type ATPase motif and a SPC metal binding sequence that were conserved among heavy-metal pumps (PIB-type ATPases). Among the 11 classes of the P-type ATPase superfamily, P1B-ATPases are responsible for transporting metals and are present in all organisms from bacteria to humans [22, 28]. Second, mutation of *dr1440* resulted in cellular accumulation of iron ions compared with wild-type cells. Third, the protein was located in the cell membrane, as demonstrated by fluorescence labelling analysis. Fourth, the *dr1440* mutant displayed a sensitive phenotype to iron ions, indicating a deficiency in iron ion export. Moreover, DR1440 mRNA and protein levels were up-regulated significantly under iron ion stress. Together, these results suggested that DR1440 was involved in the maintenance of iron homeostasis.

A high intracellular Mn/Fe ratio is correlated with the resistance to oxidative stress in *D*. *radiodurans*, and the Mn/Fe ratio of irradiation-resistant bacteria is higher than that of irradiation-sensitive bacteria, suggesting that protection of proteins from oxidative damage and maintenance of a relatively low level of intracellular iron ions are crucial to the stress-resistance of *D*. *radiodurans* [20, 29]. We previously identified a manganese efflux protein (MntE) in *D*. *radiodurans* and found that the *mntE* mutant was resistant to H_2_O_2_, ultraviolet and γ-ray radiation [20]. In the present study, we found that expression of DR1440 was up-regulated under oxidative stress, and its mutation led to the increased cell sensitivity to oxidative stress. The high intracellular concentration of iron in the DR1440 mutant resulted in increased ROS levels and protein oxidation compared with the wild-type cells, indicating that DR1440 contributed to intracellular protein protection against ROS derived from ferrous ion-mediated Fenton-reaction. *D*. *radiodurans* has several oxidation-related regulators including OxyR (DR0615) and a Fur homolog (DR0865), which are believed to regulate intracellular Mn and Fe ions [18, 19, 30]. Our previous study demonstrated that a unique diphtheria toxin repressor (DtxR) homologue (DR2539) acts as a regulator of a Fe-dependent transporter gene (*dr1219*) and a Mn transporter gene (*dr2283*) [30]. However, the exact regulation of DR1440 and co-regulation of Mn and Fe transporters to increase the intracellular Mn/Fe ratio in *D*. *radiodurans* requires further studies.

Metal binding sites vary in different classes of P-type ATPases [13]. DR1440 contains a conserved SPC motif, but not a typical CXXC motif or His-rich sequences that are found in the N-terminal metal-binding domain (NMBD) of the PIB-type ATPase from *Thermus thermophilus* [31]. A previous study demonstrated that Zn^2+^-binding of P-type ATPases depends on Cys392 and Cys394 in the CPC motif M4 [32], which is required for heavy metal transmembrane transport. Mutation of Cys in the CPC of *E*. *coli* CopA resulted in the loss of copper transport [33]. Herein, we identified S297 and C299 in the SPC motif as important residues for the function of DR1440. Further study including protein structure analysis is required to elucidate the exact roles of the SPC motif.

In conclusion, DR1440 appeared to play an important role in iron homeostasis and resistance to oxidative stress in *D*. *radiodurans*. This potential iron efflux protein may export intracellular iron ions under stress conditions to enhance cellular resistance and protect proteins against oxidative damage. Our findings not only provided new insight into the role of DR1440 in iron homeostasis and oxidative-stress resistance in *D*. *radiodurans*, but also broadened our understanding of iron transport systems in bacteria.

## Supporting information

S1 File Combined supporting information file. (DOCX)
