# Supplementary material for: DR1440 is a potential iron efflux protein involved in maintenance of iron homeostasis and resistance of Deinococcus radiodurans to oxidative stress
Source: PLoS One. 2018 Aug 14;13(8):e0202287. doi: 10.1371/journal.pone.0202287 (PMC6091924; doi:10.1371/journal.pone.0202287)

**Supporting information**

**DR1440 is a potential iron efflux protein involved in maintenance of iron homeostasis and resistance of *Deinococcus* *radiodurans* to oxidative stress**

Shang Dai ^1^, Ye Jin ^1^, Tao Li ^1^, Yulan Weng ^1^, Xiaolin Xu ^2^, Genlin Zhang ^2^, Jiulong Li ^1^, Renjiang Pang ^1^, Bing Tian ^1^*, Yuejin Hua ^1^

^1^ Key Laboratory for Nuclear-Agricultural Sciences of Chinese Ministry of Agriculture and Zhejiang Province, Institute of Nuclear-Agricultural Sciences, Zhejiang University, Hangzhou, China;

^2^ Key Laboratory for Green Processing of Chemical Engineering of Xinjiang Bingtuan, School of Chemistry and Chemical Engineering, Shihezi University, Shihezi, Xinjiang, China

^*^Corresponding author:

Email : tianbing@zju.edu.cn (BT)

**Table A. Bacterial strains and plasmids used in this study.**

| Strains and plasmids | Relevant genotype | Reference or source |
| --- | --- | --- |
| *Deinococcus radiodurans* R1 | ATCC 13939 | American Type Culture Collection (www.atcc.org) |
| Mt-1440 | *D. radiodurans dr1440* gene knock out mutant | This work |
| Mt-1440C | Mt1440 complemented with pRAD1440 | This work |
| Mt1440_wtcS297A | Mt1440 complemented with pRAD1440S297A | This work |
| Mt1440_wtcP298A | Mt1440 complemented with pRAD1440P298A | This work |
| Mt1440_wtcC299A | Mt1440 complemented with pRAD1440C299A | This work |
| Wt_pRADG | R1 complemented with pRADG | This work |
| Wt_pRADG-*dr1440* | R1 complemented with pRADG-dr1440 | This work |
|  |  |  |
| *Escherichia coli DH5a* | F-φ80 *lac* ZΔM15 Δ(lacZYA-arg F) U169 *endA*1 *recA*1 *hsdR*17(rk-,mk+) *supE*44λ- *thi*-1 *gyrA*96 *relA*1 *pho*A | TransGen |
|  |  |  |
| *Plasmids* |  |  |
| pRADZ3 | *E. coli*-*D. radiodurans* shuttle vector carrying the *lacZ* and *groEL* promoter (Ap^r^ Cm^r^) | [1] |
| pRADK | pRADZ3 derivative in which *lacZ* is replaced  with the kanamycin gene (Ap^r^Km^r^Cm^r^) | Laboratory  Stock |
| pRAD-*dr1440* | pRADK derivative in which the kanamycin gene is replaced with gene *dr1440* | This work |
| pRAD-S297A | pRAD-*dr1440* with a site mutation S297A | This work |
| pRAD-P298A | pRAD-*dr1440* with a site mutation P298A | This work |
| pRAD-C299A | pRAD-*dr1440* with a site mutation C299A | This work |
| pRADG | pRADZ3 derivative in which *lacZ* is replaced with the eGFP gene | This work |
| pRADG-*dr1440*  pMD18-T | pRADG ligated with gene dr1440  TA cloning vector with streptomycin gene | This work  Takara |

1. Meima R, Lidstrom ME. Characterization of the minimal replicon of a cryptic *Deinococcus radiodurans* SARK plasmid and development of versatile *Escherichia coli-D-radiodurans* shuttle vectors. APPL Environ Microb. 2000;66(9): 3856-3867.

**Table B. Primers used in this study^a^**

| **Primer** | **Sequence** |
| --- | --- |
| *Construction of dr 1440 mutant* |  |
| P1 | 5'-ATCAGGCCGAGCGGAATGAAAAT-3' |
| P2 | 5'-CGCGGATCCGGGGACTCCTTGCAGGAAAAG-3' |
| P3 | 5'-CCCAAGCTTCGACGCCCCGGCCCT-3' |
| P4 | 5'-CACGAGCTGCGCGAGTACGA-3' |
| P5 | 5'- GGCGCCCGGCAGCAAGAAATTC -3' |
| P6 | 5'- CGCTGCCGTTGAGGTTGACCGTG -3' |
| P_his_1 | 5'-CAAGATGACGCTGAGCCACACC-3' |
| P_his_2 | 5'-CCGGATCCTCAATGATGATGATGATGATGCGCCGTCGCCCACCATCG-3' |
| P_his_3 | 5'-CCAAGCTTACGACGCCCCGGCCCTCG-3' |
| P_his_4 | 5'-CGGCCATACGGCACGAGCTG-3' |
| streptomycin forward | 5'-CCCAAGCTTGATATCGAATTCGAGCTCG-3' |
| streptomycin reverse | 5'-CGGGATCCTTATTTGCCGACTACCTTG-3' |
|  |  |
| *Complementation of dr1440 mutant* |  |
| *dr1440*_c_ forward | 5'-GGAATTCCATATGACCGTTTCCAACCCCAC-3' |
| *dr1440*_c_ reverse | 5'-CGCGGATCCTCACGCCGTCGCCCAC-3' |
|  |  |
| *Site*-*directed mutagenesis* |  |
| *dr1440*_S297A_ forward | 5'-CTTTATGGTAGTGGCCGCGCCCTGCGCCG-3' |
| *dr1440*_S297A_ reverse | 5'-CGACGGCGCAGGGCGCGGCCACTACCATA-3' |
| *dr1440*_P298A_ forward | 5'-GTGGCCTCGCCCGCCGCCGTCGTGATTTCGA-3' |
| *dr1440*_P298A_ reverse | 5'-GTCGAAATCACGACGGCGGCGGGCGAGGCC-3' |
| *dr1440*_C299A_ forward | 5'-GTAGTGGCCTCGCCCGCCGCCGTCGTGATTTC-3' |
| *dr1440*_C299A_ reverse | 5'-GAAATCACGACGGCGGCGGGCGAGGCCACT-3' |
|  |  |
| *Real-time quantitative PCR* |  |
| *dr1343* | F: 5'-CGGCTGGTTTTCCGCATCC-3'  R: 5'-CTCGCCCCACTTGATGTTGGC-3' |
| *dr1440* | F: 5'-CGCCTGACCTGCGCTTG-3'  R: 5'-AGTTTCTTTTCGACGAACAGGC-3' |

^a^ Underlines indicate the respective restriction sites

**Fig A. Sequence alignment of DR1440 with P-type ATPase homologs**. DR1440 from *D. radiodurans*, SsZntA from *Shigella sonnei*, BsZntA from *Bacillus subtilis*, LpCopA from *Legionella pneumophila*, EcCopA from *Escherichia coli* and TtCopA from *Thermus thermophilus*. Alignment was performed using CLUSTALW software (<http://www.genome.jp/tools-bin/clustalw>). A conserved metal binding sequence SPC was indicated by dashed line box. Identical residues are shown as white letters with black background, and similar residues are shown as white letters with gray background.


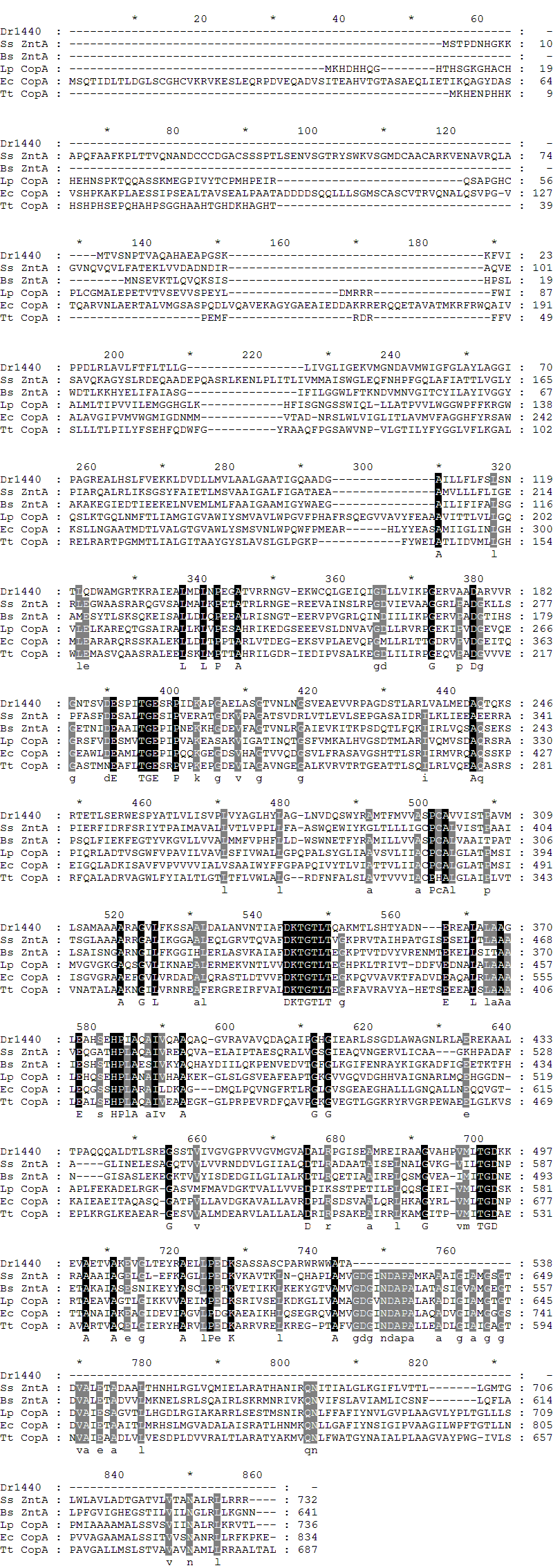


**Fig B.** **Construction of *dr1440* knock-out mutant and 6** × **His tag fused mutant.** (A) Scheme of gene mutation by homologous recombination which replaced the *dr1440* with streptomycin-resistant fragment. P1, P2, P3, P4, P5 and P6 refer to the primer pairs (Supporting information S2 Table). (B) Scheme of construction of 6 × His tag fused mutant of *dr1440* by homologous recombination which replaced the TGA (termination codon) of *dr1440* with 6 × His tag and streptomycin-resistant fragment. P_his_1, P_his_2, P_his_3, and P_his_4 refer to the primer pairs (Supporting information S2 Table). (C) PCR analysis to confirm the mutation of *dr1440*. Lane 2 and lane 4, DNA amplified using primer pairs P1 and P4 in the wild type and the mutant, respectively. The corresponding amplicon (lane 4, 1880 bp) from the mutant is 685 bp shorter than the amplicon from the wild type (lane 2, 2565 bp), indicating that *dr1440* was replaced with the streptomycin-resistance fragment. Further, an interior DNA fragment (NC_001263.1: c1444754-1445364) of the targeted gene was detected by amplification using primers P5 and P6 (lane 3 and lane 5). No products corresponding to the size of the fragment from wild type (lane 3) was observed in the mutant (lane 5), suggesting that the wild type alleles had completely replaced by streptomycin-resistance fragment in the mutant. Lane 1, Marker. (D) PCR analysis to confirm the 6 × His tag fused mutant. Lane 2 and lane 3, DNA amplified using primer pairs P_his_1 and P_his_4 in the wild type and the mutant, respectively. The corresponding amplicon (lane 2, 1034 bp) from the wild type is 918 bp shorter than the amplicon from the mutant (lane 3, 1952 bp), indicating that TGA of *dr1440* was replaced by the 6 × His tag with streptomycin-resistance fragment in the mutant. Lane 1, Marker.


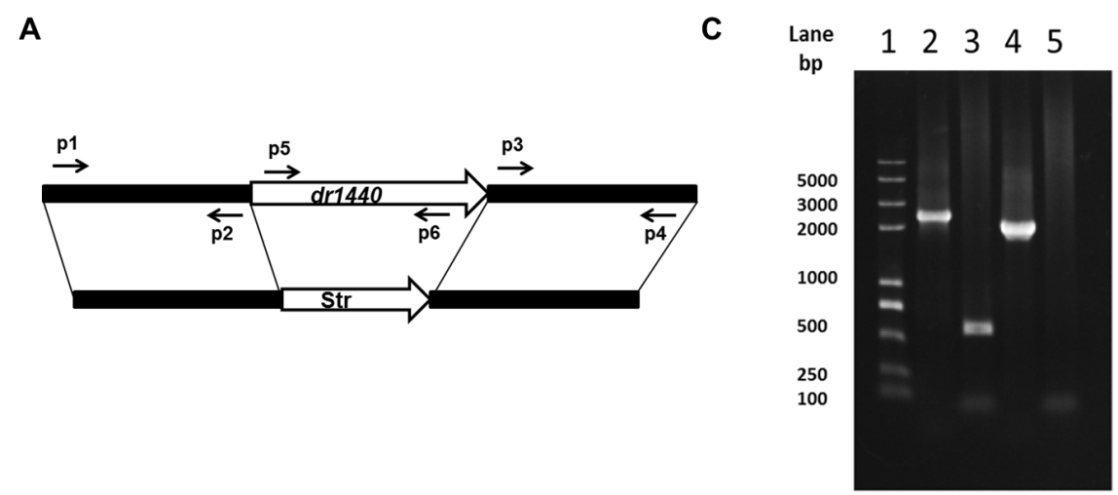


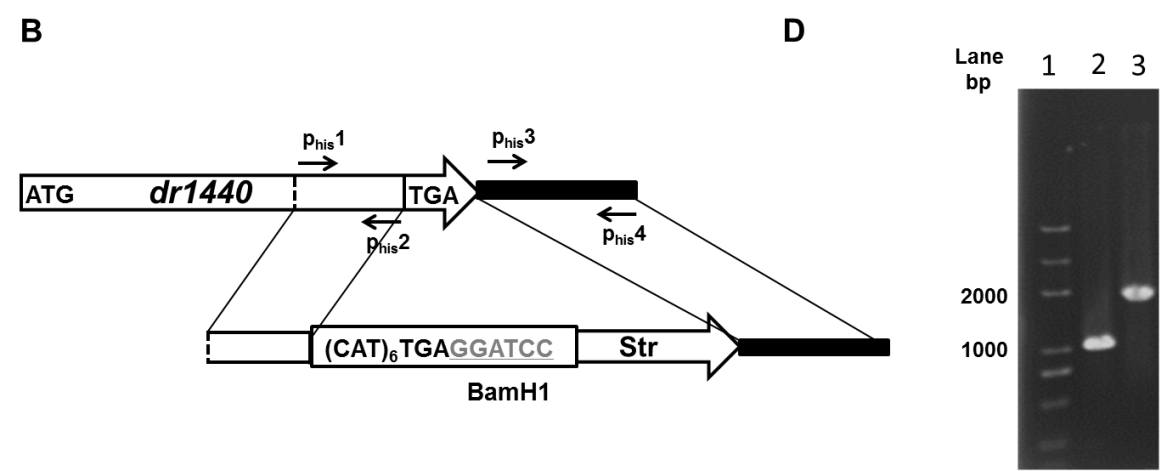


**Representative full uncropped and un-altered blot images that were used to make Fig. 2D.** Upper panel, protein expression level of DR1440 under the treatment of 1 mM Fe^2+^ or Mn^2+^, compared with untreated control. Lane 1, untreated; lane 2, treated with 1 mM MnCl_2_; lane 3, treated with 1 mM FeCl_2_; lane 4-6 is the repeat experiment for line 1-3 and were used to make Fig. 2D. A monoclonal anti-6× His mouse antibody (Proteintech, USA) was used to detect the 6 × His tag fused to the C-terminal of DR1440 (MW = 57 KD). The non-specific bands shown in the blot might come from the blot of some proteins which have similar sequence composition to the 6 × His tag. The non-specific bands can be distinguished from DR1440 in their molecular weights (MW). Bottom panel, the protein expression level of GroEL was served as an internal control. GroEL was detected by a rabbit anti-GroEL antibody (Sigma, USA) at the same treatment condition to that of DR1440. Lane 4-6 are the repeat experiments for lane 1-3 and were used to make Fig. 2D.

1 2 3 4 5 6


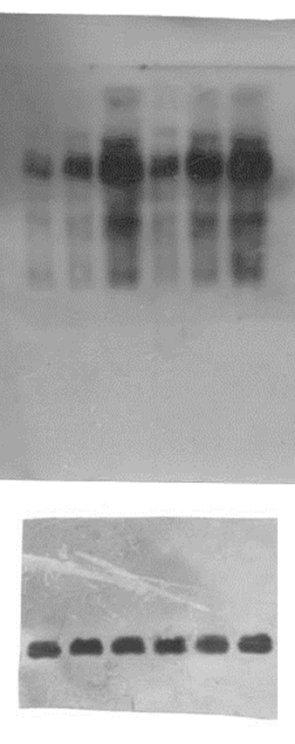


70kD

55kD

40kD

100kD

**Representative full uncropped and un-altered blot images that were used to make Fig. 4E.** Upper panel, protein expression level of DR1440 under the treatment of H_2_O_2_, compared with untreated control. Lane 1, untreated; lane 2, treated with 30 mM H_2_O_2_; lane 3, treated with 60 mM H_2_O_2_; lane 4-6 is the repeat experiment for line 1-3 and were used to make Fig. 4E. A monoclonal anti-6 × His mouse antibody (Proteintech, USA) was used to detect the 6 × His tag fused to the C-terminal of DR1440 (MW = 57 KD). The non-specific bands shown in the blot might come from the blot of some proteins which have similar sequence composition to the 6 × His tag. The non-specific bands can be distinguished from DR1440 in their molecular weights (MW). Bottom panel, the protein expression level of GroEL was served as an internal control. GroEL was detected by a rabbit anti-GroEL antibody (Sigma, USA) at the same treatment condition to that of DR1440. Lane 4-6 are the repeat experiments for lane 1-3 and were used to make Fig. 4E.

1 2 3 4 5 6


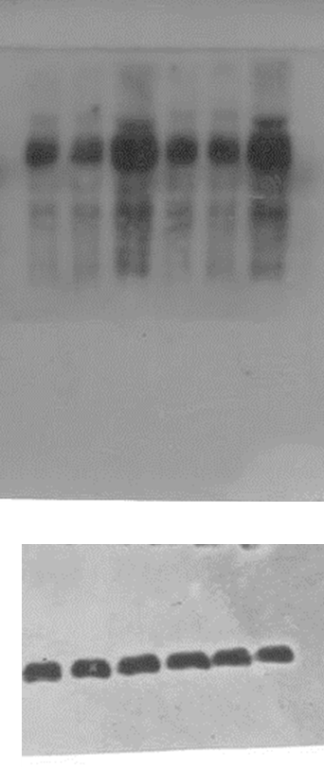


70kD

55kD

40kD

100kD

**Representative full uncropped and un-altered blot images that were used to make Fig. 5B**. Left panel, protein carbonylation levels in the wild type and Mt-1440 treated with or without 50 mM H_2_O_2_ was measured using western blot assays (OxyBlot Protein Oxidation Detection Kit, Merck Co.). M, marker; Lane 1, untreated wild type R1; Lane 2, untreated Mt-1440; Lane 3, the wild type R1 in the presence of H_2_O_2_ treatment; Lane 4, Mt-1440 in the presence of H_2_O_2_ treatment. Right panel, the protein expression level of GroEL in the respective protein samples (Lane 1-4) was detected by anti-GroEL and served as protein loading control.

M 1 2 3 4


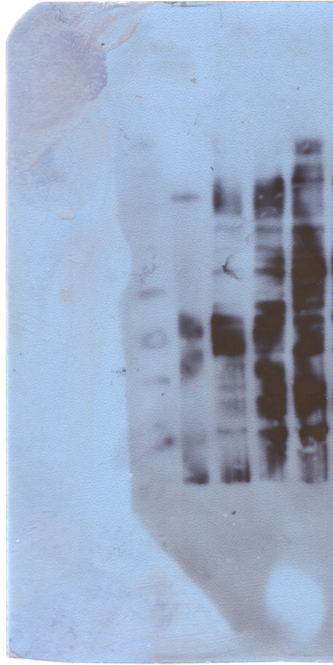

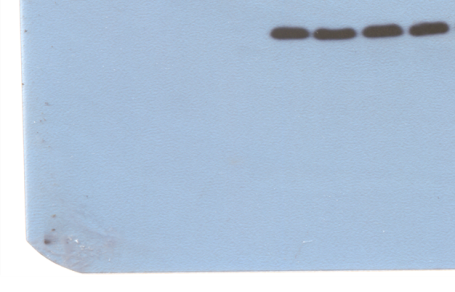

Supplement: S1 File — (DOCX) [file pone.0202287.s001.docx]
